# Southern Surgical and Gynecological Association

**Published:** 1897-01

**Authors:** 


### Society Reports.

### SOUTHERN SURGICAL AND GYNECOLOGICAL ASSOCIATION.

Ninth annual meeting, held in Nashville, Tenn., November 10, 11 and 12, 1896, under the Presidency of Dr. E. S. Lewis of New Orleans, La.

The Association met at the Nicholson House, and was called to order at 10 A. M.

The "Address of Welcome" was delivered by the Hon. John Bell Keeble, of Nashville, which was responded to by President Lewis.

"Vaginal *versus* Abdominal Section for Pus in the Pelvis." This paper was read by Dr. W. D. HAGGARD, Jr., of Nashville, in which the author recounted the transitional periods in the treatment of pus in pelvis—vaginal puncture, superseded by abdominal section and removal of pyosalpinx, total uterine castration per vaginam by the French, and through the abdomen by the American school. These operations had given way to modern vaginal section, and evacuation and drainage of all pus pockets. The abdominal route affords visual inspection of the field. The attack on morbid masses could be made with safety to visceral integrity. If pus accumulations were multiple, rupture and peritoneal soiling were inevitable; that is the supreme disadvantage of abdominal incision. The author had often seen the pelvis deluged with pus with impunity. He had also seen patients die within twelve hours from fulmanent sepsis from peritoneal contamination. The writer referred to a mortality of 18.5 per cent. in a series of collected cases of laparotomy for pus, done in five metropolitan hospitals in the last year, and asked what must it be in the "unheard-from precincts," and in the hands of the great unwashed? The abdominal method offers the best approach in tubercular inflammation of the ovaries and tubes and in small unilateral pus tubes. The author referred to the advantages of exploring the pelvis for retro-uterine tumors, inflammatory and adnexæ, by vaginal section.

The geography of pus in the pelvis in most cases makes vaginal incision extra-peritoneal, a minor procedure giving major results. No shock, no risk, no disturbance in convalescence. In prolonged sepsis from large abscesses, posterior section and drainage were a life-saving procedure. The special indications were in (1) early cases of acute suppurating salpingitis. (2) Incipient post-puerperal peritonitis. (3) Large pyosalpinx and true pelvic abscess. The first group included early gonorrheal and abortion cases. In puerperal cases, incipient peritonitis and puddles of pus in Douglas' space imperatively demanded incision. Should simple pus-letting in any of these cases not effect a cure, subsequent operation for removal of the relics of previous ravages can be done without the dangers incurred in the presence of pus. The field of vaginal section is to prevent suppuration in early cases, to anticipate it in puerperal cases, and to save life in desperate cases. Its application to the pelvic inflammatory processes and to pus in the pelvis was one of the greatest surgical triumphs of the age.

"Cholelithiasis." A paper on this subject was contributed by Dr. A. M. CARTLEDGE, of Louisville, in which the author reported several interesting cases. He dwelt upon cholecystotomy and cholecystenterostomy, pointing out the indications for each operation. He considered cholecystostomy as the only operation that was applicable to the cases cited. In his opinion there were no cases that primarily demanded cholecystenterostomy.

Dr. JAMES MCFADDEN GASTON, of Atlanta, agreed with the essayist that in ordinary cases of gall stones in the gall bladder, with obstruction of the cystic duct, the simplest procedure was to lay open the abdominal wall, attach the gall bladder to the incision, and remove the gall stones. But in a large proportion of cases of complete obstruction, he doubted whether there would be restoration of the bile through the cystic duct into the gall bladder. With reference to the comparative value of cholecystostomy and cholecystenterostomy, the two operations were applicable to entirely different conditions. No one would operate and expect benefit from a cholecystostomy except to establish drainage for the bile in a case of permanent occlusion of the common duct, and this was the only condition in which the advocates of cholecystenterostomy had ever claimed anything for it.

Dr. GEORGE BEN JOHNSTON, of Richmond, Va., spoke of the diagnosis of gall stones. He is convinced that if examinations of suspected cases of gall stones were as careful and minute as they should be, surgeons would frequently find them. It has been his experience that enlargement of the gall bladder does not always occur where a gall stone exists, but that a condition that simulated enlargement of the gall bladder frequently did exist, this condition being due to the presence of numerous dense adhesions found in the neighborhood of the gall bladder, glueing it to every tissue with which it came in contact. One thing which struck him as singular in connection with the presence of gall stones was that the size of the stone or stones seemed to make no difference in the production of symptoms. In regard to hemorrhage, it was generally admitted that in cases in which cholemia was profound, they were the ones in which we were to expect hemorrhage, and by no known method could this hemorrhage be successfully controlled. The cholemic condition seems to invite a fatal hemorrhage. The experience of operators in this field of surgery was that when cholemia was profound, hemorrhage of a fatal character was to be expected. He considers cholecystostomy a proper procedure in all cases, except in those where the obstruction was in the common duct, and could not be relieved.

Dr. W. E. B. DAVIS said surgery of the gall bladder for the removal of gall stones had given brilliant results, but there were still questions in regard to operative procedures on the ducts that were not as yet definitely settled. He did not believe the essayist referred to cholecystostomy as being the choice of operation in cases where the obstruction of the duct could not be removed; that he must have had in mind the procedure advocated by Murphy of resorting to this operation in case of gall stone in the gall bladder where there was no obstruction in the duct. Murphy resorted to cholecystenterostomy instead of cholecystostomy, and he thought the essayist did not intend to convey the idea that he would not do a cholecystenterostomy where the obstruction in the duct could not be removed. Cholemic cases were bad to operate upon. Perhaps in not more than 5 or 6 per cent. of the cases was the obstruction found in the common duct. Some years ago the author made experiments which conclusively showed that the surgeon could incise the duct and drain with gauze, without peritonitis following. A paper on this subject was read by him before the American Medical Association in 1892, since which time he had done further experimental work in which sutures were not used after the stone was removed from the duct, and while several of the cases were already very nearly dead from profound cholemia, and eventually did die, yet in the cases in which this method was resorted to, the abdominal cavity was walled off, and peritonitis did not result.

Dr. F. W. McRae, of Atlanta, reported a case in which there were repeated attacks of colic, with profound cholemia. An operation was undertaken, with the idea that the obstruction was in the common duct, and that there were stones in the gall bladder. On opening the abdomen, in the presence of several physicians, the liver was found much enlarged and reaching almost to the umbilicus. Instead of finding the gall bladder enlarged, he found a fibrous cord not larger than his index finger. The common duct, from disuse, was reduced to a mere cord. A calculus was found in the hepatic duct extending up into the transverse fissure of the liver. He did not know what to do for a case like this, and, after consultation with his colleagues, closed the abdomen. The patient died five days later from exhaustion. If anything could be done for such patients, he would like to know it.

"Mental Complications following Surgical Operations." Dr. John T. Wilson, of Sherman, Texas, read this paper. He said the subject of mental disorders produced by or following surgical operations had not been discussed to any great extent, and until within the past two years only a passing notice had been given to it. It was a strange fact that while surgical operations would sometimes cause serious mental disturbance, on the other hand, those same operations would sometimes cure them; especially was this the case with some melancholiacs. Many females, laboring under attacks of melancholia, caused by some disease of the genitals, had been cured when relieved of the physical—by operation; others had been much improved, and yet some had received no benefit. The question might very properly be asked, why a surgical operation should produce an attack of insanity. This could no more be answered in every case satisfactorily, than could the question why some persons became insane from the many other causes to which it was attributed, for in most cases the mental complications were a surprise, and no good reason could be given why they should follow. In others, however, a logical explanation might be had. If the patient was a high-strung, nervous individual, easily excited, unable to bear pain, the great and increasing dread of the anesthetic, the operation, or both, would so affect him that he would lose control of the will power and the explosion would come after the operation and reaction from the anesthetic. In many of these cases, probably a majority, there was an hereditary taint or a strong neurotic tendency.

The author quoted Mairet, who thinks (1) that it is in those individuals who are predisposed by heredity, or other grave causes—alcoholism, infectious diseases, etc., that surgical operations give rise to insanity. (2) In the constituent elements of an operation that might act on the brain, the two most important ones were the anesthetic and the degree of surgical traumatism with its after effects, of which disturbed nutrition plays a very important part. (3) When predisposition also is considerable, the anesthetic alone may produce insanity, or it may result even after minor operations. It is, of course, necessary to take into consideration the mental state of the patient prior to the operation, especially in those graver ones where questions of life or death are frequently involved.

"Splitting the Capsule for the Relief of Nephralgia." Dr. George Ben Johnston, of Richmond, Va., read a paper with this heading, in which he drew the following conclusions: (1) Nephralgia is not always associated with a demonstrable lesion. (2) When other evidences of kidney diseases are wanting, the pain is due to a too tight capsule. (3) Nephralgia may, and frequently does, simulate symptoms of gross tissue changes or mechanical irritants. (4) Where severe and persistent pain in the kidney exists without other evidences of renal disease, exploratory operation is indicated. (5) When inspection, palpation, and needle puncture fail to disclose a condition sufficient to account for the pain, the capsule should be freely opened.

"Uretero–Ureteral Anastomosis." Dr. J. Wesley Bovee, of Washington, D. C., read a paper on this subject, and reported an interesting case. The author dwelt at length upon the literature of the subject, quoting from the contributions to the surgery of the ureters by Van Hook, Fenger, Kelly and Cabot in this country, and the classical works of Glantenay, Liaudet, Tuffier, and others in Europe. He drew the following conclusions: (1) Uretero-ureteral anastomosis, whenever possible, is far preferable to any other form of ureteral grafting, to nephrectomy, and to ligation of the ureter. (3) It should be done preferably by lateral implantation, or by oblique end-to-end anastomosis, though the tranverse end-to-end, or the end-in-end methods, may be safely employed. (4) That constrictions of the calibre of the ureter do not usually follow attempts at suturing in closure of complete transverse section of the duct. (5) That nephrectomy for transverse injuries of the ureter *per se* is an unjustifiable operation. (6) That simple ligation of the ureter to produce extinction of the function of the kidney is too uncertain to justify its practice. (7) That drainage is not necessary if the wound be perfectly closed and the tissues are septic.

"The Treatment of Pregnancy and Labor Complicated by Fibroid Tumors of the Uterus." Dr. Henry D. Fry, of Washington, D. C., read this paper. He advanced two propositions: First, that the production of abortion is unjustifiable; second, that labors presenting serious difficulty to delivery are best treated by abdominal section and removal of the child and tumor. By maintaining this position the interests of the mother are not relegated to second place. While saving the lives of many infants, the maternal mortality will also be diminished. After making a few brief remarks on the natural history of fibroid tumors complicating the pregnant state and reporting a few cases that had come under his care. he considered the treatment.

Dr. HOWARD A. KELLY agreed with the conclusions of the essayist. There was a tendency on the part of the profession to interfere too much in cases of pregnancy complicated by fibroid tumors of the uterus. He had been called in consultation to see a number of such cases, but the indications were not such in some of them as to warrant the induction of premature labor. In many instances a consultation had been the means of postponing operative interference. When fibroid tumors complicating pregnancy were situated in the upper part of the uterine body, unless large and multiple, they were comparatively unimportant. If situated in the lower part of the uterus, and it is found that as pregnancy advances that they can be pushed up, this should be done in order that the labor may proceed naturally. On three occasions he had opened the abdomen and had done a myomectomy for tumors complicating pregnancy, the women subsequently going to full term and being delivered normally.

Dr. E. S. LEWIS said it often falls to the lot of some physicians to meet with a series of anomalous cases, such as those that had been reported by the essayist, whilst other physicians, with probably quite as large experience, would pass through life without meeting some of the complications that had been mentioned. During an experience extending over thirty-four years he had never met with a fibroid tumor which justified interference before labor; that is, a fibroid occupying the lower segment of the uterus and impinging upon the pelvic cavity. Within the past year he had delivered two women having large fibroids. In one case, where pregnancy supervened, after suspension of menstruation for two months, he was unable for several months to determine the existence of the pregnancy. The uterus then reached above the umbilicus, but the woman was found pregnant and was delivered at full term with forceps, but with no extraordinary difficulty. The other woman had an abdominal tumor the size of a six months fœtus. Although she had been married a number of years, she was about forty when she became pregnant. The tumor occupied the upper portion of the body of the uterus, but she was delivered without the use of instruments. He could conceive that in a case of fibroid situated in the broad ligaments or occupying the lower segment of the uterus, seriously impinging upon the cavity of the uterus, hysterectomy would be inevitable, but it has been his fortune to escape such cases.

"Uterine Drainage as a Factor in the Prevention and Relief of Pelvic Inflammation," by Dr. R. R. Kime, of Atlanta, who drew the following conclusions: (1) A uterine tampon is not a true drain, and even obstructs drainage in many cases.

(2) Capillary drainage is secured by carrying a strip of gauze up into the uterine cavity, not packing it, and then it drains for a few hours only. (3) Gauze can not even act as a capillary drain when either end or center is constricted, or when coated with mucus. (4) Gauze, when saturated with serum, unless it contains an antiseptic, forms a hotbed for germ development. (5) Never tampon the uterus in puerperal septic infection, except to check the hemorrhage. (6) The good effect of a gauze tampon in cases of endrometis and after abortion is not due to drainage, but to its effects as a tampon, *i. e.*, checking hemorrhage, stimulating uterine contractions, prolonging medication to the endometrium, and acting as a surgical dressing. (7) The uterine drainage tube is the most essential factor in the treatment of puerperal infection and the best method of securing drainage when demanded in other diseased conditions of the uterus. (8) It will save more lives, relieve and prevent more pelvic complications than any other factor at our command.

Dr. W. E. PARKER, of New Orleans, read a paper on "Gunshot Wounds of the Abdomen," and reported thirteen cases, with six recoveries. In his paper he made the statement that he believed, in the hands of men skilled in abdominal work, seventy-five per cent. of cases of wounds of the small intestine should recover if the cases were seen early, the prognosis being better in this class of cases than in any other. He advised an early and rapid operation in all cases.

In conclusion, he made the following general statements: The diagnosis is generally easy, but, when in doubt, he advised enlarging the wound or probing. In doubtful cases he was inclined to attach much importance to pain referred to the umbilicus as a symptom. He stated that he had never seen a case where this symptom was not present. There is frequently but little shock when grave symptoms are present, and when symptoms of it are present the trouble is generally hemorrhage and not shock. Senn's gas test was not used in any of these cases, and he spoke of it as being unnecessary in at least a majority of cases, uncertain in the hands of those not skilled in its use, and making it more difficult to replace the intestines after sewing the wounds.

As to the technique he said; (1) Unless the wound is well to one side, it is best to make a median incision, and it should be long enough to enable the operator to make a thorough examination of the abdominal contents. (2) The whole intestinal canal should, as a rule, be examined. (3) All peritoneal wounds should be sutured with silk Lembert sutures. Intestinal wounds should, other things being equal, be sewn in the long axis of the bowel. (4) If the liver is wounded, better results are obtained from packing than from suturing it. (5) If the kidney has been wounded, it is best to suture the peritoneal wound and treat the kidney extra-peritoneally, if necessary. Of course, he did not refer to those where the laceration and hemorrhage are so great that it is necessary to remove the kidney at once. (6) Drainage, except in late cases, is not necessary, if all hemorrhage has been stopped. (7) Cases in which the intestines can not be sutured without great risk of obstruction should be resected. (8) While enough time should be taken to do the work thoroughly, no time should be wasted. (9) Unless the bullet can be felt, search should not be made for it, as it causes unnecessary delay. (10) The superficial wound should be closed with silk-worm gut or silver wire, and the author believes that a single suture should include the skin, abdominal walls and peritoneum.

*Prognosis.* (1) The sooner the patient is operated upon, the better the prognosis. (2) Those cases that have been reported in a series, including all cases, have shown a mortality of about sixty-two per cent. The prognosis is best in cases of wounds of the small intestines, and he believes that seventy-five per cent. of the cases will recover if seen early. By early he means in the first two or three hours. (3) We all know that alcoholics stand all surgical work badly, and yet most of these patients have been drinking before they come under our care. The prognosis in non-alcoholics would be better than in alcoholics. (4) If the stomach and intestines are empty, the prognosis is usually improved by this fact.

*After treatment.* While not favoring drugging these patients, strychnine and other stimulants should be given hypodermatically, if necessary. Especially should strychnine and alcohol in some form be given to alcoholics. Much depends on starting these patients well. If restless after the operation or suffering, small doses of morphine should be given. If the stomach is quiet and has not been injured, small amounts of water and Ducro's elixir can safely be given at the end of twenty-four hours and small quantities of milk or some light broth can be given. If the stomach has been injured, the feeding should be per rectum. The diet should be liquid for at least two weeks. If there is shock, with the clammy sweat that is sometimes seen, atropine, gr. $\frac{1}{60}$, should be given every three hours, as may be necessary. When shall we give a purgative? This is one of the most important questions that we will be called upon to decide. If we give a purgative too soon our stitches may pull out, and if we wait adhesions may form and give us trouble. The bowels of these patients will usually act by themselves about the end of the fifth or beginning of the sixth day. If they do not, a mild purgative, assisted by an enema, should be given about the end of the sixth day, or on the morning of the seventh. As a rule, these patients should be kept in bed for at least two and a half weeks.

Dr. HOWARD A. KELLY offered the following resolution, which was unanimously adopted:

*Resolved*, That it is the sense of all the members of the Southern Surgical and Gynecological Association that in gunshot wounds penetrating the abdominal cavity, the proper routine procedure is to make an immediate exploratory incision.

Dr. Parker said, in closing, that the late Dr. Miles, in his first series of cases, reported thirteen cases, the percentage of recovery being nearly forty per cent. He had operated on probably twenty additional cases before his death, and the percentage of recovery was very much better than in the first series. As to the medico-legal aspects of this subject, all surgeons should advocate the early opening of the abdomen, and if some fellow practitioner should get into trouble as a result of it, the profession should stand together and support him.

Dr. L. S. MCMURTRY, of Louisville, read a paper entitled "The Evolution and Perfection of the Aseptic Surgical Technique." The author cited cases where surgeons of world-wide reputation had infected their patients through some imperfection in the aseptic surgical technique, and said that the subject deserves more study and attention at the hands of operative surgeons than had heretofore been given to it. So far as instruments, dressings, etc., are concerned, surgeons had an absolute guarantee against sepsis in sterilization by heat; but when it came to the operative field, the hands of the operator and his assistants, they were reduced to mechanical and chemical methods of asepsis, which were certainly far less efficacious and reliable than sterilization by heat. Everything that comes in contact with the field of operation in the form of instruments and dressings is exposed to heat at a boiling temperature, hence the patient was safe against septic infection, but so much could not be said for the hands of the surgeon and those of his assistants in the field of operation.

"The President's Address." This was delivered by Dr. E. S. Lewis, of New Orleans. Reference was made to the brilliant achievements of the masters of the art of surgery who had passed away, and of the galaxy of shining lights who had followed after, who had created an era in the medical history of this country for all future time. How can we wonder that the "magnificent records obtained by experts have proved alluring temptations to the inexperienced and ambitious," and led to abuses which have left a blot on the fair page of abdominal surgery. As a representative body of the surgeons and gynecologists of the South, it should condemn the reckless and thoughtless plunging in this delicate and difficult work, without knowledge, fitness, or preparation. The statistics of the skilled, who had learned to minimize risk and cope with difficulties, should not serve as an argument with the inexperienced to secure subjects. The responsibility of human life should not be ignored in the craving and struggle for notoriety or fame.

With regard to the relative merits of the abdominal and vaginal operations for the removal of the ovaries and tubes, or of the uterus with the appendages, President Lewis said that divergent opinions are entertained and heated discussions have arisen. For the vaginal method it is claimed less shock is produced, better drainage is obtained, the abdominal walls are not weakened, and the extirpation of the uterus removes a menacing source of infection and of physical and nervous disturbance. For the abdominal operation, rapidity of execution is contended with increased security to adjacent organs, and facility of repair when injured, as the structures are always in view. The removal of the uterus is also condemned as complicating and unwarrantable unless justified by the state of the organ. In the modified vaginal method, as practiced by Doyen and others, the uterus is not necessarily sacrificed, nor a sound ovary and tube. It is in touch with the conservative movement of the day, and is in marked contrast with the ultra-radical operation of Pean.

"Memorial Address on Dr. Paul F. Eve." This was delivered by Dr. Richard Douglas, of Nashville, in which he said a retrospect of the lives of great men inspired us with spirit of emulation and indicated to the ambitious mind the paths to fame. Professor Paul F. Eve had three distinguishing characteristics—energy, consistency of purpose and extreme modesty, and upon them he built for himself an everlasting reputation, and secured an imperishable place in the temple of fame. It is not alone as surgeon and teacher that his reputation rests. As a contributor to current medical literature he was a conspicuous authority. In military surgery he was without a peer. His experience in Poland had engrafted a taste for the work which, unfortunately, in later years, as one of the chief surgeons of the Confederacy, he had more than ample opportunity to gratify. As the result of his observation and work during the War of Secession, he recorded many valuable facts which the surgeons of to-day would do well to ponder. As a lithotomist Dr. Eve was pre-eminent. While his preference was for the bilateral method, yet he was not wedded to it, and appreciated the many advantages of the suprapubic operation, and often practiced it, not, however, with the same success that he achieved by perineal section. Thoroughness characterized every undertaking of his life. When the great and good life of Dr. Eve came to an end, suddenly but peacefully, on November 3d, 1877, he had reached more than his three-score years and ten, and, dying, left behind him a name that was destined to live on in surgery through many generations.

A paper on "The Relations of Tubercular Diathesis to its Local Manifestations" was read by Dr. J. McFadden Gaston, of Atlanta. He said that in considering the various forms in which tuberculosis shows itself in different structures, there must be an underlying element pervading the whole organism, which results from a general determination of the secretion. Whether there is a predisposition to the development of tuberculosis in certain parts or organs in advance of any constitutional disease or not, this change occurs in connection with the general impairment of the vital forces which characterizes the tubercular diathesis. While most recent authorities do not make a distinction between scrofula and tuberculosis, there is a fundamental difference in their general and local development. We have different characteristics of tuberculosis when it involves separate organs and structures of the body in a distinctly circumscribed form, or is defined as miliary tubercle in different structures, and yet the dyscrasia which marks the lymphatics under the designation of scrofula differs materially from any of the varieties of tuberculosis heretofore recognized. Dr. Gaston touched briefly on the causes of tuberculosis, and reference was made to the papers that were presented before the last meeting of the American Surgical Association on important tubercular lesions. The presence of a condition recognized as a tubercular diathesis corresponds in some respects with the cachexia of carcinomatous tumors, and is held by many to be hereditary. There has been quite a revolution in the opinions of those best versed in the pathology of tuberculosis as to the transmission of this disease from parent to child, and also in regard to the communicability from one individual to another by ordinary contact in social relations. It is fair to conclude that great caution should be observed in putting restraints upon the marriage of those suffering with pulmonary consumption, and the association of those laboring under this disease should be limited as far as practicable. Finally, the predisposition to tuberculosis can not be relieved by a surgical operation upon the diseased structures, but must be corrected by remedial agencies acting through the absorbent and secretory organs.

"The Rational Treatment of the Diseased Appendix by Operation." Dr. A. V. L Brokaw, of St. Louis, read a paper with this title. He said the question had been vigorously discussed, is appendicitis a surgical disease at all times, or only surgical at times? He wished to be put on record as favoring the first proposition. He was aware that some ultra so-called conservative practitioners claimed that the surgeon who advocated the removal of the appendix in every case when diseased, was a dangerous faddist, an extremist suffering from an inoculation of the *operativus bacillus.* He earnestly advocated early operation as soon as the diagnosis was made. Always operate when there is even a slight chance of saving a life, regardless of damage to statistics. Invariably operation should be insisted upon in the recurrent cases. With the knowledge of this dread disease, evolved from the mortuary chambers and the treacherous clinical course in a considerable percentage of cases, why should the rational treatment of all cases be other than by prompt surgery?

"Report of Cases of Appendicitis." Dr. James A. Goggans, of Alexander City, Ala., followed with a paper on this subject. From the fact that physicians generally take the stand that operative interference in appendicitis is called for only in exceptional instances where the disease advances to suppuration, gangrene and perforation, makes the treatment of appendicitis a never-ceasing controversy, hence his excuse for reporting a few illustrative cases that had come under his observation, hoping thereby to add what he could to harmonize the difference between the physician and surgeon on this, the most frequent and important intra-abdominal lesion, in his opinion, of the present day. The main point at issue between the physician and the surgeon in the treatment of appendicitis depended much on a perfect diagnosis. This, too, accounted in a measure for their differences of opinion as to where the medical treatment should end, and where the surgical treatment should begin. According to his experience in the management of this affection, there is only one course to pursue, namely, to remove the appendix just as soon as the diagnosis has been made. Usually he defers the operation until the bowels have been evacuated by first administering a few small doses of calomel, followed by a saline purge.

Dr. CHARLES P. NOBLE said the safest general rule was to operate as soon as a diagnosis of appendicitis was made. It was impossible to differentiate the cases which would recover from a primary attack from those that would die.

DR. M. C. MCGANNON, of Nashville, recalled one case of appendicitis, a boy, where the temperature arose to 105°. The patient was delirious. Upon opening the abdomen the appendix was found to be black but not perforated. It was easily removed, and the boy made a prompt recovery. He believes in many cases that, if the physician waits and watches for distinct symptoms before operating, patients will die.

Dr. A. M. CARTLEDGE said the diagnosis was the only problem that practitioners were especially concerned with, together with the proper technique in the execution of the operation. The more he operates, the more he is inclined to believe we should operate on every operable case as soon as the diagnosis has been made. Mistakes were made by waiting and watching for symptoms to manifest themselves. Very few, if any, surgeons could tell when an appendix had ruptured.

Dr. GEORGE BEN JOHNSTON, said, for the sake of statistics, operations for appendicitis should be divided into two classes. First, those which are performed for recurrent attacks of the disease, and those which are employed for relief of the severer varieties where perforation has occurred, or will take place where there is pus present. If the surgeon is to operate upon recurrent cases, it is better for him to do so between the attacks in order that he may choose his time for operation. While there were cases of this disease that recovered without treatment, the best results were obtained by surgical interference.

Dr. W. E. B. DAVIS thought there were few cases of appendicitis that gave rise to general peritonitis in which the surgeon was called and could do any good. Frequently the surgeon was called too late. Even though the family physician recognized the condition, it was not an easy matter to persuade the patient to be operated on within the first twenty-four hours, and unless these cases were treated surgically within twenty-four hours or thirty-six hours very few of them could be saved. All cases of severe attacks of the disease, in which pain is intense, if seen the first day and consent is obtained, should be operated on. In all cases where there is a second attack operative measures should be resorted to.

Dr. H. M. HUNTER, of Union Springs, Ala., reported an interesting case of "Compound Comminuted Fracture of the Radius and Ulna Near the Wrist Joint." He had been unable to find a similar case on record in the literature of fractures. There were three points with regard to this case: First, that he was unaware of a similar fracture being reported. Second, he had never read nor heard of the method he had described to reduce the fracture of the forearm. Third he had never seen nor read of such perfect results as were obtained in this case, the wrist having perfect motion, and there is absolutely no interference with supination and pronation.

Dr. N. P. DANDRIDGE, of Cincinnati, reported a case of trans-peritoneal ligature of the external iliac artery for inguinal aneurism, in which he removed the aneurismal sac.

In the discussion, Dr. W. E. B. Davis also reported a case of ligation of the common iliac for aneurism of the external iliac, which was followed by an excellent result.

The following officers were elected:

President—Dr. George Ben Johnston, of Richmond, Va.

First Vice-President—Dr. F. W. McRae, of Atlanta, Ga.

Second Vice-President—Dr. W. E. Parker, of New Orleans, La.

Secretary—Dr. W. E. B. Davis, of Birmingham, Ala.

Treasurer—Dr. A. M. Cartledge, of Louisville, Ky.

The Association then adjourned to meet in St. Louis, Mo., the second Tuesday in November, 1897.

---

### IT PAYS TO TAKE YOUR HOME MEDICAL JOURNAL.

Many doctors have no idea of the number of inquiries which come to the local medical journal regarding the physicians living within the territory covered by such a periodical. The doctor hundreds of miles away from the home office of the journal, may be as anxiously inquired about as the one living in the building where it is published. Life insurance companies, accident companies, bond and security companies, business houses and banks, corporations of all kinds, and private individuals and firms write for information. The journal is looked upon as a sort of general bureau, which is drawn upon for information from all quarters. There is on the part of the journal a presumption that something is wrong with the medical man who does not take his home journal, and such *rara avis* needs to be very well known by the periodical to have its warmest endorsement. Again, if the doctor pays his subscriptions promptly, it helps to throw light upon the question so often asked about his financial standing.—*Medical Sentinel.*

